# When cocrystals become radical-activable

**DOI:** 10.1093/nsr/nwaf170

**Published:** 2025-04-26

**Authors:** Miaomiao Xue, Yinjuan Huang, Qichun Zhang

**Affiliations:** Department of Materials Science and Engineering, Department of Chemistry, Center of Super-Diamond and Advanced Films (COSDAF) and Hong Kong Institute of Clean Energy, City University of Hong Kong, China; State Key Laboratory of Porous Metal Materials, Shaanxi International Research Center for Soft Matter, School of Materials Science and Engineering, Xi'an Jiaotong University, China; Department of Materials Science and Engineering, Department of Chemistry, Center of Super-Diamond and Advanced Films (COSDAF) and Hong Kong Institute of Clean Energy, City University of Hong Kong, China

Organic cocrystals, formed by at least two different organic small molecules via diverse forces (e.g. π–π interactions, hydrogen/halogen bonding and

**Figure 1.**
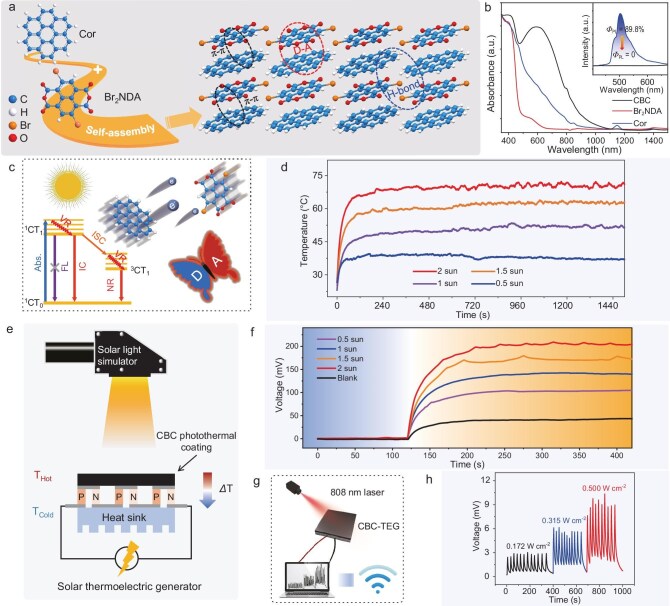
(a) Molecular structures of Cor, Br_2_NDA, and crystal stacking of the cocrystal. (b) Absorption spectra and photoluminescence spectra. (c) Jablonski diagram. (d) Temperature changes of CBC coating by irradiation of minic solar source (808 nm laser with a power density of 0.300 W cm^−2^). (e) Schematic diagram of CBC-TEG. (f) The output voltage of CBC-TEG under mimic solar source (808 nm laser with a power density of 0.300 W cm^−2^) with different intensities. (g) Schematic diagram of the information transmission. (h) The output voltage changes of CBC-TEG. Reproduced from ref. [5] with permission.

charge transfer (CT)), can allow researchers to engineer their bandgaps and tune their optical/electrical properties for various applications [1,2]. In particular, the donor-to-acceptor electron delocalization in organic CT cocrystals can induce new orbital hybridization, promoting the formation of narrow bandgap and red-shifted absorption, which can boost the notable photothermal conversion [3,4]. Furthermore, if open-shell radicals (half-filled bands originated from the π-orbital overlap) are realized in cocrystals, the system becomes radical-activable, and can display more efficient light absorption and non-radiative recombination. This factor is very important for improving photothermal conversion as well as enhancing efficient energy transformation. Recent research published in *National Science Review* [5] by Zhuo and co-workers provides an excellent example to affirm this.

In this research [5], Zhuo *et al.* developed an organic radical-incorporating CT cocrystal (CBC) by choosing an open-shell radical of 2,6-dibromonaphthalene-1,4,5,8-tetracarboxylic dianhydride (Br_2_NDA) as the electron acceptor, and using coronene (Cor) as the electron donor (Fig. 1a). Owing to the remarkable electron affinity of radicals, Br_2_NDA can easily accept electrons from Cor, leading to a high degree of electron delocalization and strong CT interaction. This process further contributes to the reduced energy bandgap with a strong near-infrared absorption close to 1100 nm as well as non-radiative recombination (Fig. 1b), resulting in a high photothermal conversion efficiency of 67.2% for the cocrystals (Fig. 1d). The femtosecond transient absorption (Fs-TA) results proved that the ultrafast non-radiative process of excited states (Fig. 1c), including internal conversion (IC), intersystem crossing (ISC), and charge dissociation to the ground state caused by the radicals, promote highly efficient energy conversion from solar to thermal energy.

Furthermore, a photothermal ink was fabricated using the CBC cocrystal as the active component and the transparent resin was applied as the matrix, which can be easily coated on a thermoelectric generator as a cost-effective light absorber to form a high-performance solar thermoelectric generator (STEG) (i.e. CBC-thermoelectric generator (TEG)) (Fig. 1e). Impressively, a high photothermal temperature of 70.3°C can be achieved, and the as-prepared STEG can output a voltage of 209 mV under irradiation of a simulated solar source with an intensity of 2 suns (Fig. 1d and f). Moreover, the STEG was also endowed with the capabilities of non-contact and long-distance real-time information conversion, exhibiting great potential in self-powered optoelectronics (Fig. 1g and h).

In summary, this paper reports an effective strategy to fabricate organic radical-activable CT cocrystals with greatly reduced bandgap and rapid non-radiative dynamic pathways via using radical molecules as the electron acceptor, which promotes superior photothermal conversion. This strategy provides innovative guidance for developing novel organic radical-activable co-crystals for advanced applications in photothermal conversion as well as in thermoelectric generators.
